# Force-Sensor-Based Analysis of the Effects of a Six-Week Plyometric Training Program on the Speed, Strength, and Balance Ability on Hard and Soft Surfaces of Adolescent Female Basketball Players

**DOI:** 10.3390/s26030758

**Published:** 2026-01-23

**Authors:** Guopeng You, Bo Li, Shaocong Zhao

**Affiliations:** 1 Department of Physical Education, Xiamen University of Technology, Xiamen 361000, China; youguopeng@xmut.end.cn; 2 School of Physical Education and Sport Science, Fujian Normal University, Fuzhou 350007, China; libo@fjnu.edu.com

**Keywords:** plyometric training, balance, adolescent female athletes, soft surface, neuromuscular control, explosive power

## Abstract

This study investigated the effects of 6 weeks of plyometric training (PT) performed on soft (unstable) and hard (stable) surfaces compared with conventional training on the balance, explosive power, and muscle strength of adolescent female basketball players. The participants were randomly assigned to three groups: soft-surface PT (n = 14), hard-surface PT (n = 14), and conventional training (n = 14). Performance outcomes included 30 m sprint time, vertical jump height, plantar flexion and dorsiflexion maximal voluntary isometric contraction (MVIC) torque, Y-balance dynamic balance, and center of pressure-based static balance. Ground reaction forces, MVIC torques, and balance parameters were measured using high-precision force sensors to ensure accurate quantification of biomechanical performance. Statistical analyses were performed using two-way repeated-measures ANOVA with post hoc comparisons to evaluate group × time interaction effects across all outcome variables. Results demonstrated that soft- and hard-surface PT significantly improved sprint performance, vertical jump height, and plantar flexion MVIC torque compared with conventional training, while dorsiflexion MVIC increased similarly across all the groups. Notably, soft-surface training elicited greater enhancements in vertical jump height, dynamic balance (posteromedial and posterolateral directions), and static balance under single- and double-leg eyes-closed conditions. The findings suggest that PT on an unstable surface provides unique advantages in optimizing neuromuscular control and postural stability beyond those achieved with stable-surface or conventional training. Thus, soft-surface PT may serve as an effective adjunct to traditional conditioning programs, enhancing sport-specific explosive power and balance. These results provide practical guidance for designing evidence-based and individualized training interventions to improve performance and reduce injury risk among adolescent female basketball athletes.

## 1. Introduction

Basketball require athletes to possess well-developed postural control and balance ability [1,2]. For adolescent female basketball players, who are in a critical stage of physical development, improving balance ability is particularly essential not only for enhancing sport performance but also for mitigating the elevated risk of lower limb injuries, [3]. However, current youth training programs frequently prioritize strength and skill development while placing insufficient emphasis on balance-oriented interventions, resulting in a potential gap in motor control development.

Balance is a multifactorial neuromuscular function that involves the visual, vestibular, and proprioceptive systems working together to maintain stability under static and dynamic conditions [4]. Given its strong dependence on coordinated neuromuscular responses, balance can be substantially influenced by training modalities that target rapid force production, proprioceptive acuity, and sensorimotor integration. In this context, plyometric training (PT)—a modality that leverages the stretch–shortening cycle to enhance neuromuscular performance—has been widely applied to improve lower-limb power, coordination, and agility [5,6]. Although traditional PT is frequently performed on stable surfaces (e.g., hardwood floors), recent evidence suggests that executing plyometric movements on unstable surfaces (e.g., foam pads, sand, or balance platforms) may offer superior proprioceptive stimulation and postural control demands [7,8]. The added instability is believed to amplify afferent feedback from muscle spindles and Golgi tendon organs, thereby necessitating rapid modulation of spinal reflex excitability (e.g., H-reflex) and supraspinal control to maintain equilibrium [9]. However, other studies have argued that excessive surface instability may disperse force application, reduce peak force output, or limit power development during explosive movements. This potential trade-off between enhanced postural demand and force production efficiency underscores the need for empirical comparison of stable and unstable surface PT effects [10]. Additionally, available studies have predominantly focused on adult or male athletes [11,12]. Crucially, studies on this topic have yet to concurrently employ high-precision biomechanical analysis to assess these adaptations in a vulnerable and understudied population. Specifically, the distinct physiological and biomechanical traits of adolescent females—including lower baseline muscle strength, greater joint laxity, and specific neuromuscular control patterns—necessitate population-focused investigation [13]. Research investigating how different support surfaces influence neuromuscular and balance adaptations in adolescent females is particularly lacking, representing a critical gap that the present study aims to address.

With the advancement of wearable and platform-based sensor technologies, such as force plates and inertial measurement units, biomechanical assessments have become increasingly precise and objective. These tools enabling the quantification of subtle variations in postural sway, ground reaction forces, and movement symmetry [14,15]. Moreover, they provide valuable insight into the neuromuscular adaptations induced by PT under different surface conditions, thereby offering a more comprehensive understanding of sensorimotor control mechanisms in youth athletes [15].

Therefore, the present study aimed to investigate the effects of 6 weeks of PT performed under different support conditions (stable versus unstable surfaces) on the speed, strength, and balance ability of adolescent female basketball players. Dynamic balance was assessed using the Y-balance test, while static balance was evaluated with center-of-pressure measures during single-leg stance. In addition, lower limb explosive performance was recorded to examine the concurrent effects on physical fitness. We hypothesized that PT performed on a soft, unstable surface would produce greater improvements in static and dynamic balance compared with training on a hard, stable surface, due to increased proprioceptive demands and neuromuscular activation. Regarding explosive strength, we expected that both soft- and hard-surface PT would enhance performance relative to conventional training; moreover the unstable surface was anticipated to induce additional neuromuscular coordination gains. The findings of the current study are expected to offer practical insights into evidence-based conditioning strategies for adolescent female athletes and contribute to an increased understanding of how surface properties modulate the efficacy of PT in youth sport development.

## 2. Materials and Methods

### 2.1. Participants

An a priori power analysis was conducted using G*Power 3.1.9.7 (University of Düsseldorf, Düsseldorf, Germany) for a repeated-measures ANOVA with three groups and two time points. Based on previous findings on surface-dependent plyometric adaptations [11], we used an expected effect size of f = 0.40, α = 0.05, power = 0.80, and an assumed correlation of 0.5 between repeated measures. The analysis indicated that at least 21 participants were required. To ensure sufficient power and accommodate potential attrition, 42 participants were ultimately recruited from Changzhi Sports School in Changzhi City, Shanxi Province, China.

The inclusion and exclusion criteria for participant recruitment was shown in Table 1. All participants were screened for contraindications prior to enrollment. All participants were competitive adolescent basketball players who trained with their school team 5.0 ± 0.6 sessions per week, each session lasting 90 ± 10 min, and participated in organized competition (regional level). None reported regular (structured) plyometric training as part of their weekly program in the 6 months preceding enrollment; occasional informal jumping drills during team practice were common.

**Table 1:** Inclusion and Exclusion Criteria for Participant Recruitment.

Category	Criteria
Inclusion criteria	Female basketball players aged 13–16 years
	No lower limb injuries in the past 3 months
	Right leg-dominant
Exclusion criteria	Neurological or vestibular disorders
	Cardiovascular/systemic contraindications to exercise
	Medications affecting neuromuscular control

The participants were instructed to abstain from strenuous physical activities within 24 h prior to testing, obtain at least 8 h of sleep and avoid staying up late the night before testing, and refrain from consuming any beverages or medications that may affect neural function (e.g., alcohol, cola, coffee, tea, or similar substances) within 4 h before the test. Before the experiment, all the participants were fully informed about the study’s objectives and procedures and signed a written informed consent form. The study protocol was reviewed and approved by the Institutional Ethics Committee of Changzhi Medical College, with approval number RT2023041 on 13 July 2023.

### 2.2. Experimental Procedures

Participants engaged only in their routine school basketball activities during the intervention period, with no additional technical, tactical, or external training permitted.

After screening, participants were stratified by age and performance level. Randomization was performed by an independent researcher (not involved in enrollment) using a computer-generated random number table. Participants were allocated into groups (n = 14 per group) through sequentially numbered, sealed, opaque envelopes to ensure concealment of the assignment. While blinding of participants and training coaches was unfeasible due to the nature of the training intervention, all baseline and post-intervention testing, as well as subsequent data analysis, were conducted by researchers blinded to the group assignments to prevent detection bias. Participants were stratified into either the PT group on a soft surface (n = 14), PT group on a hard surface (n = 14), or conventional training groups (n = 14).

Training surface specification: Plyometric training was performed on two clearly defined support surfaces. The soft (unstable) surface consisted of AIREX^®^ Balance Pads (closed-cell AIREX^®^ foam; 50 × 41 × 6 cm; AIREX AG, Sins, Switzerland; product: Balance Pad/Balance Pad Elite), which are manufactured from a proprietary closed-cell foam designed for balance and rehabilitation use. The pads have been characterized as a relatively low-density, yielding foam (density ≈ 38–39 kg·m^−3^), providing a compliant and perturbing support commonly used in balance and plyometric interventions [11,12]. The hard (stable) surface consisted of a competition-grade hardwood gym floor (maple, 25 mm thickness) over a concrete subfloor, which exhibits negligible compliance relative to the Airex pad. During training sessions, exercises designated for the soft-surface group were executed directly on single AIREX pads placed on the gym floor. The pads were moveable but were positioned flat and unstacked for all participants and sessions, and the same set of pads was used throughout the 6-week intervention to preserve a consistent instability profile.

**Figure 1:**
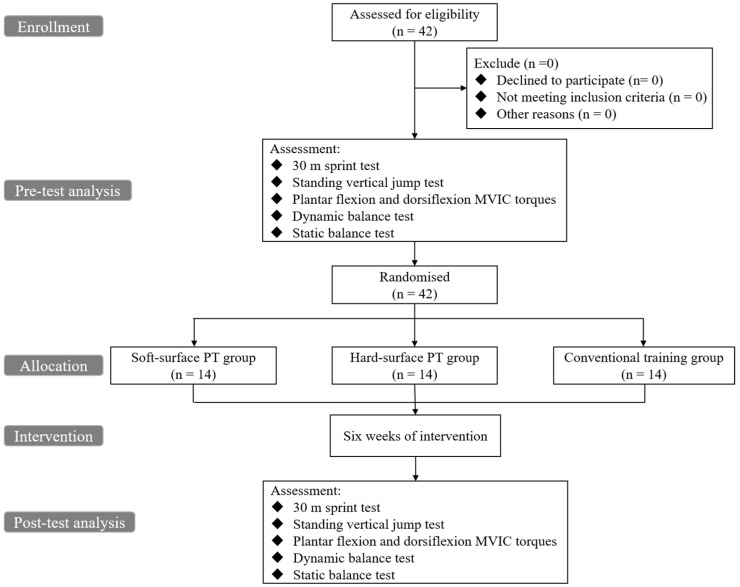
Flowchart of study.

All instructions, familiarization procedures, and safety briefings were provided individually to each participant to ensure consistent understanding of the testing and training protocols. Prior to the intervention, all participants completed a two-week preparatory phase consisting of weekly training sessions focused on fundamental movement patterns. As illustrated in Figure 1, baseline testing was conducted during the first visit. After a standardized 10 min structured dynamic warm-up and a brief rest (three minutes), the participants performed a series of pre-intervention assessments. This warm-up consisted of two phases: a 5-min general warm-up (light jogging and comprehensive joint mobility exercises) followed by a 5-min specific dynamic warm-up. The dynamic warm-up targeted the lower extremity and included sequential movements such as walking lunges, controlled high-knees, butt kicks, and lateral shuffles, with each exercise performed for 20 m or 30 s. Static stretching was strictly avoided prior to the main training intervention. Pre-intervention assessments included a 30 m sprint test, standing vertical jump, maximal voluntary isometric contraction (MVIC) torque of plantar flexion and dorsiflexion, dynamic balance, and static balance. The order of tests was randomized, with rest intervals of approximately 3–5 min between different test to minimize carryover fatigue. Each test was repeated three times, While the best attempt reflects maximal physiological capacity, the mean value was used for subsequent analysis to provide a representative measure of the participants’ performance consistency, considering the potential for high intra-individual variability in adolescent athletes. Data were screened for potential outliers solely as a data quality control measure to identify and remove results stemming from equipment failure or severe protocol violations (e.g., timing errors or false starts), ensuring all valid maximal efforts were preserved. Rest intervals of approximately 1–2 min were provided between the same test.

All pretests were conducted on the Friday prior to the start of intervention. The training interventions were then implemented on a thrice-weekly basis (Monday, Wednesday, and Friday) for 6 weeks. All training sessions were supervised by certified strength and conditioning staff. Coaches provided standardized verbal cues (e.g., soft landing, neutral trunk, proper knee alignment) and immediate visual feedback to ensure correct jumping and landing mechanics. Technique quality was monitored continuously, and repetitions not meeting safety or form criteria were repeated. All post-testing assessments were conducted on the Sunday following the final training session, ensuring that each participant had more than 24 h of rest before post-intervention measurements. After each training and testing session, a 15-min recovery activity is conducted, which includes static stretching and other exercises. Each test in the pre-test and post-test was conducted by the same, well-trained researchers.

Because all participants were adolescent females, menstrual-cycle status was recorded at baseline using a brief self-report checklist. However, testing and training sessions were scheduled on fixed weekly days for logistical consistency, and therefore were not aligned to specific menstrual-cycle phases. As a result, potential hormonal variation across the testing period could not be fully controlled.

### 2.3. PT

The PT program was designed using a progressive overload approach, with gradually increasing intensity and volume over a 6 weeks period. The training consisted of five exercises that targeted lower limb explosive power, performed three times per week (Monday, Wednesday, and Friday). The following exercises were included: (1) squat jumps, (2) single-leg step-up jumps onto a 40 cm box (alternating legs), (3) double-leg box jumps onto 40 cm or 60 cm boxes, (4) forward hurdle jumps over a 60 cm barrier, and (5) vertical reach jumps. The detailed weekly training plan is presented in Table 2.

To ensure training quality and safety, two qualified strength and conditioning coaches supervised all sessions. Training intensity was strictly monitored as maximal voluntary effort. Participants were instructed to perform every jump with maximum explosiveness, focusing on minimizing ground contact time and maximizing jump height. Progression was controlled through both volume and technical complexity. The volume gradually increased from 112 to 168 foots contacts per week. Exercise intensity progressed by increasing the box height. Crucially, this progression was contingent upon technical competency. Participants were only allowed to increase box height or complexity when they could demonstrate correct takeoff and soft-landing mechanics without valgus collapse.

**Table 2:** Plyometric training program.

Week	Exercise	Sets × Repetition	Rest Interval
Week 1	Squat Jumps	3 × 8	3 min
	Single-leg Box Jumps (40 cm box)	3 × 8	
	Double-leg Box Jumps (40 cm box)	3 × 12	
	Forward Hurdle Jumps (60 cm height)	3 × 8	
	Vertical Reach Jumps	3 × 8	
Week 2	Squat Jumps	3 × 10	3 min
	Single-leg Box Jumps (40 cm box)	3 × 8	
	Double-leg Box Jumps (40 cm box)	3 × 12	
	Forward Hurdle Jumps (60 cm height)	3 × 10	
	Vertical Reach Jumps	3 × 10	
Week 3	Squat Jumps	3 × 12	3 min
	Single-leg Box Jumps (40 cm box)	3 × 8	
	Double-leg Box Jumps (40 cm box)	3 × 12	
	Forward Hurdle Jumps (60 cm height)	3 × 10	
	Vertical Reach Jumps	3 × 12	
Week 4	Squat Jumps	3 × 12	3 min
	Single-leg Box Jumps (40 cm box)	3 × 8	
	Double-leg Box Jumps (60 cm box)	3 × 12	
	Forward Hurdle Jumps (60 cm height)	3 × 12	
	Vertical Reach Jumps	3 × 12	
Week 5	Squat Jumps	3 × 12	3 min
	Single-leg Box Jumps (40 cm box)	3 × 10	
	Double-leg Box Jumps (60 cm box)	3 × 12	
	Forward Hurdle Jumps (60 cm height)	3 × 12	
	Vertical Reach Jumps	3 × 12	
Week 6	Squat Jumps	3 × 12	3 min
	Single-leg Box Jumps (40 cm box)	3 × 10	
	Double-leg Box Jumps (60 cm box)	3 × 12	
	Forward Hurdle Jumps (60 cm height)	3 × 12	
	Vertical Reach Jumps	3 × 12	

### 2.4. Conventional Lower Limb Training

The conventional lower limb training program included the following exercises: (1) squats, (2) stretching exercises, (3) single-leg squats, (4) push-ups, and (5) basic jump training. Each exercise was performed in four sets of fifteen repetitions, with a rest interval of 90 s between sets and 3 min between different exercises.

Exercises were performed with body mass resistance, focusing on movement quality and muscle control. For non-ballistic exercises (e.g., squats, push-ups), participants maintained a controlled tempo (approximately 2 s eccentric and 1 s concentric) to maximize motor unit recruitment stability [16]. For the basic jump training, participants performed sub-maximal jumps focusing on landing mechanics rather than maximal height. Progression was controlled by increasing movement quality and minimizing rest intervals within the prescribed range, ensuring that the total training density remained consistent with the plyometric groups.

### 2.5. Data Collection

#### 2.5.1. The 30 m Sprint Test

Sprint times were recorded using dual infrared timing gates (Brower Timing Systems, Draper, UT, USA) with 0.01 s accuracy. The timing gates were positioned at approximately hip height (90 cm above the ground) at start and finish lines. Participants were instructed to sprint from 1 m behind the start line, starting from a static standing start position. Participants were further instructed to begin sprinting with their self-selected rhythm. Verbal encouragement was provided throughout each maximal sprint. All sprinting activities were completed outdoors on field turf, with participants wearing cleats.

#### 2.5.2. Standing Vertical Jump Test

The participants stood approximately 0.5 m behind the force platform (Model: 9287C, Kistler, Winterthur, Switzerland) equipped with integrated force sensors and waited for the tester’s verbal cue. Force data were acquired at a high sampling frequency of 1000 Hz. This system was calibrated and verified using known standards on 21 March 2023. Upon hearing the command “ready,” the participant would step forward onto the force platform and positioned both feet parallel on the two plates, with hands placed on the hips and the torso upright. At the instruction of the tester, the participant would perform a countermovement, followed by an explosive vertical jump by using both legs simultaneously.

During the jumping task, the participants were instructed to maintain core engagement, keep the trunk upright, and avoid any lateral body rotation. The tester verbally encouraged each participant to jump as high as possible during every trial. Three trials were performed, with a 2 min rest interval between each trial. High-frequency kinetic data collected by the embedded force sensors were used to derive the velocity–time profile through double integration of the ground reaction force, and jump height was calculated using the impulse–momentum method [17].

#### 2.5.3. Plantar Flexion and Dorsiflexion MVIC Torques

Plantar flexion and dorsiflexion MVIC torques were assessed with the participants in the prone position. The right knee and foot were secured in the anatomical neutral position (starting position). The MVIC torque of the ankle plantar flexors and dorsiflexors was measured using a CON-TREX isokinetic dynamometer (CMV AG, Bergdietikon, Switzerland) equipped with high-precision torque and angle sensors. The dynamometer’s torque sensor has a full measurement range of 0–500 Nm and a sensitivity of 0.1\Nm. The contraction performed was strictly isometric (static), not dynamic. Participants were instructed to gradually increase their force to their maximal effort and sustain the contraction for 3 s, with a 1 min rest interval between trials to prevent fatigue. The peak torque value recorded during this sustained isometric effort was used for subsequent analysis.

#### 2.5.4. Dynamic Balance Test

Dynamic balance was assessed using the Y-balance test protocol, thereby obtaining the reach distances and times in the anterior, posteromedial, and posterolateral directions. The participants stood barefoot on their left (supporting) leg on the test platform, with the toes aligned at the starting line. While maintaining the single-leg stance, the participant was instructed to reach as far as possible in the anterior, posteromedial, and posterolateral directions by sliding the reach indicator with the non-stance foot.

During the test, the participants were required to place both hands on their hips, keep their gaze forward, and avoid using the reaching foot for support or touching the ground. Three valid trials were recorded in each direction. To account for individual differences in limb length, reach distances were normalized as a percentage of leg length. Leg length was measured from the anterior superior iliac spine to the distal tip of the medial malleolus on the dominant side.

#### 2.5.5. Static Balance Test

Static balance was assessed using the Super Balance system (Acmeway, Beijing, China) in order to obtain the mean displacement and mean velocity of center of pressure in the anteroposterior (AP) and mediolateral (ML) direction. The participants stood on the platform with their feet shoulder-width apart, hands on their hips, and eyes directed forward. The test protocol included four conditions: (1) double-leg stance with eyes open, (2) double-leg stance with eyes closed, (3) single-leg stance (dominant leg) with eyes open, and (4) single-leg stance (dominant leg) with eyes closed.

Each double-leg stance trial lasted for 30 s, while each single-leg stance trial lasted for 10 s. During double-leg stance, the participants were instructed to keep both feet flat on the platform without lifting them and maintain their hands on their hips throughout the test. During single-leg stance, the non-stance foot was required to remain off the ground, with the knee slightly flexed and the foot held behind the stance leg without contacting it. Participants were instructed not to let the non-stance foot touch the stance leg, the ground, or any external object throughout the test. Any inadvertent contact or loss of position resulted in the trial being repeated. A 30 s rest interval was provided between each test condition to prevent fatigue.

### 2.6. Statistics

All statistical analyses were performed using SPSS 25.0 (SPSS Inc., Chicago, IL, USA). Prior to formal analysis, the Shapiro–Wilk test was applied to assess the normality of each variable within groups. Only data that met the assumption of normality were subjected to parametric testing.

To evaluate the effects of the intervention, a two-way repeated-measures ANOVA (Group × Time) was applied directly to all normally distributed variables. When significant main effects or interactions were detected, Bonferroni-adjusted post hoc tests were performed. If baseline differences had been present for any variable, an ANCOVA with baseline values as covariates would have been implemented; however, no such imbalance was observed. To estimate the effect sizes of the ANOVA models and post hoc analyses, we determined the partial eta-square ($\eta_{p}^{2}$). Cohen’s d was used as the effect size (ES) metric. The interpretation thresholds were defined as follows: <0.20 = trivial, 0.20–0.59 = small, 0.60–1.19 = moderate, 1.20–1.99 = large, 2.00–3.99 = very large, and ≥4.00 = extremely large [18].

For variables not meeting the normality assumption, the Friedman test was used to assess within-group changes, and the Kruskal–Wallis test was additionally conducted to compare between-group differences at each time point. Statistical significance was set at *p* < 0.05.

## 3. Results

**Figure 2:**
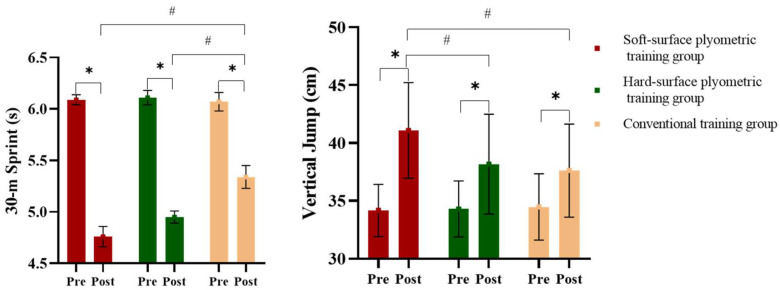
Effects of plyometric training under different support surfaces on 30 m sprint time and vertical jump height (mean ± SD). * Indicates a significant difference between pre- and post-training within the same support surface condition (*p* < 0.05); # indicates a significant difference between groups after training (*p* < 0.05).

**Figure 3:**
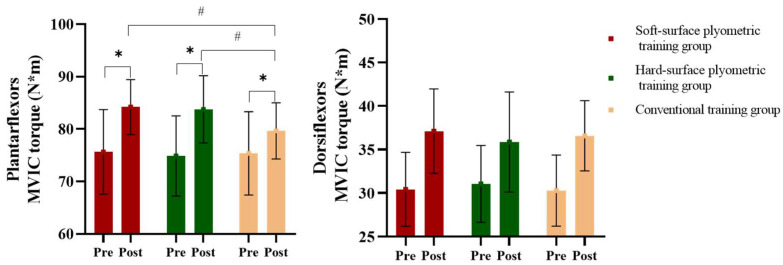
Effects of plyometric training under different support surfaces on plantarflexion and dorsiflexion MVIC torque (mean ± SD). * Indicates a significant difference between pre- and post-training within the same support surface condition (*p* < 0.05); # indicates a significant difference between groups after training (*p* < 0.05).

All 42 participants successfully completed the 6-week training intervention, with complete data sets available for analysis. Training logs confirmed full adherence to the prescribed volume and progression for each session, and no adverse events, injuries, or safety concerns were reported throughout the study. Initial testing demonstrated no significant baseline differences between groups in anthropometric characteristics, Speed, Strength, or Balance Ability (*p* > 0.05) (Table 3, Table 4 and Table 5, Figure 2 and Figure 3).

**Table 3:** Basic Information of the Participants.

Characteristics	Soft-Surface PT Group	Hard-Surface PT Group	Conventional Training Group	*p* Value
N	14	14	14	
Age (years)	15.3 ± 1.2	14.7 ± 1.5	16.1 ± 1.4	0.51
Height (cm)	161.3 ± 6.4	160.7 ± 7.6	163.8 ± 5.6	0.19
Weight (kg)	54.5 ± 6.2	53.2 ± 4.5	55.6 ± 7.1	0.63
Years of training	7.2 ± 1.5	7.1 ± 1.3	7.1 ± 1.4	0.65

### 3.1. Effects of PT on 30 m Sprint Time and Vertical Jump Height

As shown in Figure 2, 6 weeks of PT resulted in significant interaction effects between group and time on 30 m sprint time (*F*_(1,13)_ = 7.15, *p* < 0.001, $\eta_{p}^{2}$ = 0.42, ES = 0.74) and vertical jump height (*F*_(1,13)_ = 6.47, *p* < 0.001, $\eta_{p}^{2}$ = 0.38, ES = 0.81). Significant major effects of intervention condition and time were also observed.

For 30 m sprint performance, post hoc analyses indicated that sprint times were significantly decreased in all the three groups compared with their pretraining values, with the two PT groups demonstrating significantly greater improvements than the conventional training group.

For vertical jump performance, post hoc analyses demonstrated significant increases in jump height across all the groups. However, the greatest improvement was observed in the soft-surface PT group, followed by the hard-surface PT group and then the conventional training group.

### 3.2. Effects of PT on Plantar Flexion and Dorsiflexion MVIC Torques

The two-way ANOVA revealed a significant interaction between group and time for plantar flexion MVIC torque (*F*_(1,13)_ = 5.49, *p* = 0.012, $\eta_{p}^{2}$ = 0.48, ES = 0.73), but not for dorsiflexion MVIC torque (*F*_(1,13)_ = 3.42, *p* = 0.105, $\eta_{p}^{2}$ = 0.18, ES = 0.65). Post hoc analyses indicated that plantar flexion MVIC torque after the intervention was significantly higher than at baseline in the soft- and hard-surface PT groups, and higher than that of the post-intervention conventional training group. Significant major effects of time (*F*_(1,13)_ = 17.24, *p* < 0.001, $\eta_{p}^{2}$ = 0.61, ES = 0.78) and group (*F*_(1,13)_ = 8.41, *p* = 0.016, $\eta_{p}^{2}$ = 0.47, ES = 0.63) were also observed for plantar flexion MVIC torque. By contrast, only a major effect of time was found for dorsiflexion MVIC torque, indicating that dorsiflexion MVIC torque significantly increased across all three intervention protocols (Figure 3).

### 3.3. Effects of PT on Dynamic Balance

As indicated in Table 4, the two-way ANOVA revealed no significant group × time interactions in reach distances across the three directions (anterior, posteromedial, and posterolateral) of the Y-balance test. However, significant group × time interactions were observed in completion time for all three directions (*F*_(1,13)_ = 10.54, *p* = 0.032, $\eta_{p}^{2}$ = 0.36, ES = 0.71 for anterior direction; *F*_(1,13)_ = 9.68, *p* = 0.024, $\eta_{p}^{2}$ = 0.43, ES = 0.84 for posteromedial direction; *F*_(1,13)_ = 12.17, *p* = 0.037, $\eta_{p}^{2}$ = 051, ES = 0.86 for posterolateral direction). Post hoc analyses indicated that for the anterior direction, the soft-surface and hard-surface PT groups required significantly less time to complete the task compared with the conventional training group, with no significant difference between the two plyometric groups. In addition, all three groups exhibited significant reductions in completion time from pre- to post-intervention.

For the posteromedial direction, both PT groups completed the task at significantly less time than the conventional training group. Compared with the baseline, significant reductions in completion time were observed in both plyometric groups, with the soft-surface PT group demonstrating greater improvement (shorter time) than the hard-surface PT group.

Similarly, in the posterolateral direction, the soft-surface and hard-surface PT groups required significantly less time than the conventional training group. Significant pre-to-post reductions in completion time were observed across all the three groups, with the soft-surface group outperforming the hard-surface group.

**Table 4:** Effects of Plyometric training on the distance and the duration of the movement-phase in Y-balance test (Mean ± SD).

	Direction	Intervention	Soft-Surface Plyometric Training Group	Hard-Surface Plyometric Training Group	Conventional Training Group	*p*-Value
Reach distance (%)	Anterior	Pre	65.2 ± 4.8	64.7 ± 4.6	65.5 ± 4.7	0.348
		Post	66.8 ± 5.7	66.2 ± 6.4	66.4 ± 5.5	
	Posteromedial	Pre	116.5 ± 7.3	116.1 ± 9.0	116.8 ± 7.8	0.326
		Post	118.3 ± 10.7	118.1 ± 11.7	118.5 ± 10.5	
	Posterolateral	Pre	131.7 ± 6.8	130.5 ± 5.6	130.9 ± 4.5	0.203
		Post	133.1 ± 7.2	131.6 ± 7.1	130.7 ± 6.8	
Time (s)	Anterior	Pre	5.12 ± 1.35 ^a^	5.24 ± 1.02 ^a^	5.07 ± 1.27 ^a^	0.032
		Post	3.11 ± 1.23 ^b^	3.36 ± 1.47 ^b^	4.75 ± 1.37 ^c^	
	Posteromedial	Pre	7.54 ± 2.32 ^a^	7.46 ± 2.44 ^a^	7.51 ± 3.01 ^a^	0.024
		Post	5.61 ± 2.41 ^b^	6.02 ± 2.57 ^c^	7.14 ± 2.64 ^a^	
	Posterolateral	Pre	5.18 ± 1.84 ^a^	5.31 ± 1.62 ^a^	5.27 ± 1.77 ^a^	0.037
		Post	3.21 ± 1.24 ^b^	3.95 ± 1.45 ^c^	4.67 ± 1.51 ^d^	

Different letters denote significant differences between groups or time points, while identical letters indicate no significant difference (repeated-measures ANOVA).

### 3.4. Effects of PT on Static Balance

Two-way ANOVA showed no significant group × time interactions for static balance under bipedal and unipedal eyes-open conditions. By contrast, significant group × time interactions were observed for mean AP displacement(*F*_(1,13)_ = 9.47, *p* = 0.042, $\eta_{p}^{2}$ = 0.61, ES = 0.86) and mean ML (*F*_(1,13)_ = 8.81, *p* = 0.035, $\eta_{p}^{2}$ = 0.57, ES = 0.78) displacement under bipedal eyes-closed condition. Post hoc comparisons indicated that from pre- to post-intervention, the soft-surface and hard-surface PT groups exhibited significant reductions in AP and ML displacements. Post-intervention values in both PT groups were significantly lower than those in the conventional training group, with no difference between the two PT groups.

Under unipedal eyes-closed condition, significant group × time interactions were found for mean displacement (*F*_(1,13)_ = 12.42, *p* = 0.038, $\eta_{p}^{2}$ = 0.47, ES = 0.86 for AP; *F*_(1,13)_ = 10.71, *p* = 0.027, $\eta_{p}^{2}$ = 0.63, ES = 0.87 for ML) and mean velocity (*F*_(1,13)_ = 8.74, *p* = 0.005, $\eta_{p}^{2}$ = 0.55, ES = 0.82 for AP; *F*_(1,13)_ = 7.63, *p* = 0.018, $\eta_{p}^{2}$ = 0.48, ES = 0.86 for ML). Post hoc tests showed that both PT groups exhibited significant pre-to-post reductions in displacement and velocity. Post-intervention values were lower than those of the conventional group. Moreover, the soft-surface PT group demonstrated greater improvements (smaller displacement and velocity) than the hard-surface PT group (Table 5).

**Table 5:** The effect of Plyometric training on the static balance (Mean ± SD).

	Parameters	Intervention	Soft-Surface Plyometric Training Group	Hard-Surface Plyometric Training Group	Conventional Training Group	*p*-Value
Bipedal stance with eyes open	Mean AP displacement (mm)	Pre	12.41 ± 4.11	11.92 ± 5.64	12.75 ± 4.34	0.605
		Post	10.37 ± 3.72	11.03 ± 4.67	11.64 ± 3.85	
	Mean ML displacement (mm)	Pre	6.37 ± 4.68	6.45 ± 3.57	6.13 ± 4.21	0.476
		Post	5.51 ± 4.23	5.68 ± 4.71	5.59 ± 4.67	
	Mean AP velocity (mm/s)	Pre	8.67 ± 1.50	8.52 ± 1.37	8.62 ± 1.43	0.308
		Post	8.41 ± 1.28	8.27 ± 1.33	8.34 ± 1.37	
	Mean ML velocity (mm/s)	Pre	6.57 ± 1.21	6.41 ± 1.13	6.52 ± 1.37	0.679
		Post	6.42 ± 1.52	6.53 ± 1.27	6.44 ± 1.23	
Bipedal stance with eyes closed	Mean AP displacement (mm)	Pre	23.61 ± 12.31 ^a^	24.13 ± 11.75 ^a^	23.54 ± 13.84 ^a^	0.042
		Post	19.62 ± 13.39 ^b^	20.41 ± 12.97 ^b^	22.05 ± 12.75 ^a^	
	Mean ML displacement (mm)	Pre	6.61 ± 2.80 ^a^	6.37 ± 2.64 ^a^	6.58 ± 2.37 ^a^	0.035
		Post	4.17 ± 1.94 ^b^	5.34 ± 2.03 ^b^	6.08 ± 2.11 ^a^	
	Mean AP velocity (mm/s)	Pre	9.38 ± 1.38	9.49 ± 2.17	9.35 ± 1.78	0.561
		Post	9.12 ± 1.61	9.26 ± 1.57	9.38 ± 1.86	
	Mean ML velocity (mm/s)	Pre	7.13 ± 1.12	6.98 ± 1.07	7.08 ± 1.26	0.673
		Post	6.63 ± 1.35	6.77 ± 1.68	6.94 ± 1.34	
Unipedal stance with eyes open	Mean AP displacement (mm)	Pre	3.68 ± 2.81	3.62 ± 2.37	3.55 ± 2.64	0.516
		Post	3.71 ± 3.54	3.84 ± 2.34	3.86 ± 2.67	
	Mean ML displacement (mm)	Pre	108.37 ± 15.76	107.34 ± 13.85	108.83 ± 14.51	0.157
		Post	107.27 ± 14.84	107.56 ± 12.38	107.18 ± 16.48	
	Mean AP velocity (mm/s)	Pre	28.45 ± 5.47	29.35 ± 6.78	28.57 ± 5.86	0.574
		Post	28.16 ± 4.33	28.76 ± 6.47	28.49 ± 5.67	
	Mean ML velocity (mm/s)	Pre	32.36 ± 7.58	31.84 ± 8.67	32.82 ± 7.88	0.482
		Post	30.48 ± 8.07	30.87 ± 7.94	31.07 ± 6.84	
Unipedal stance with eyes closed	Mean AP displacement (mm)	Pre	16.41 ± 5.38 ^a^	15.87 ± 6.12 ^a^	16.38 ± 5.76 ^a^	0.038
		Post	11.52 ± 4.67 ^b^	13.36 ± 5.65 ^c^	15.93 ± 5.17 ^a^	
	Mean ML displacement (mm)	Pre	108.15 ± 16.34 ^a^	107.67 ± 15.94 ^a^	108.37 ± 16.25 ^a^	0.029
		Post	97.75 ± 15.29 ^b^	100.46 ± 17.38 ^c^	106.18 ± 15.08 ^a^	
	Mean AP velocity (mm/s)	Pre	67.75 ± 15.16 ^a^	68.19 ± 13.75 ^a^	67.83 ± 14.86 ^a^	0.005
		Post	52.18 ± 13.76 ^b^	58.53 ± 14.68 ^c^	65.28 ± 13.57 ^a^	
	Mean ML velocity (mm/s)	Pre	65.48 ± 16.42 ^a^	65.97 ± 17.23 ^a^	66.07 ± 17.12 ^a^	0.018
		Post	51.16 ± 15.23 ^b^	58.54 ± 16.85 ^c^	65.48 ± 15.67 ^a^	

Different letters denote significant differences between groups or time points, while identical letters indicate no significant difference (repeated-measures ANOVA). AP, anteroposterior; ML, mediolateral.

## 4. Discussion

The current study investigated the effects of 6 weeks of PT performed on soft and hard surfaces compared with conventional training on balance performance. It further explored the underlying mechanisms associated with muscle strength and motor function. Results showed that plyometric training on a soft surface produced greater improvements than conventional training across multiple indicators, whereas plyometric training on a hard surface improved several outcomes but did not significantly outperform conventional training for vertical jump; soft-surface PT yielded the most pronounced gains in balance and neuromuscular control. High-precision force sensors were integrated to continuously monitor during training and testing sessions, providing reliable biomechanical evidence to validate these outcomes. These findings highlight the critical role of surface characteristics in modulating training adaptations and offer empirical guidance for designing sport-specific training programs for adolescent female basketball players, with implications for performance enhancement and injury prevention. Compared with previous plyometric training studies, which rarely incorporated surface-dependent biomechanical analysis, the present study provides one of the first sensor-based evaluations of neuromuscular and balance adaptations across different support surfaces in adolescent female athletes. This helps fill an important gap in the literature where the interaction between surface properties and neuromechanical performance has been insufficiently explored.

After six weeks of intervention, all groups showed significant reductions in 30 m sprint time and increases in vertical jump height, with both PT groups outperforming the conventional training group. Notably, participants in the soft-surface PT group achieved greater improvements in vertical jump height than those in the hard-surface group, consistent with the principles of neuromuscular adaptation. As a classic “rapid eccentric–concentric contraction” training modality, PT enhances the storage and release efficiency of elastic potential energy, accelerates motor unit recruitment, improves synchronization of muscle fiber activation, and consequently, enhances lower limb explosive power and movement speed [19]. These superior performance gains suggest more efficient elastic energy utilization and faster force transmission during take-off. The inherent instability of a soft surface imposes higher demands on neuromuscular regulation during training. The additional proprioceptive stimulation provided by an unstable surface facilitates the synchronized activation of motor units and promotes faster neural adjustments to dynamic postural changes [20]. This adaptation improves feedforward and feedback motor control strategies [21]. From a practical perspective, the magnitude of these improvements exceeds mere statistical significance. The soft-surface group demonstrated a mean increase in vertical jump height of approximately 6.9 cm. In competitive basketball, such an improvement is meaningful, potentially translating to a decisive advantage in rebounding, shot blocking, and shooting over defenders. Similarly, the reduction in 30 m sprint time implies enhanced court transition capabilities. Consequently, compared with hard-surface training, soft-surface PT provides distinct measurable advantages in improving neuromuscular coordination and force production efficiency, offering a more targeted approach to developing sport-specific explosive power in adolescent basketball players [11].

The muscle strength results further revealed that soft- and hard-surface PT significantly increased plantar flexion MVIC torque, with greater improvements than conventional training. However, dorsiflexion MVIC torque increased significantly across all the groups, and PT did not produce additional benefits beyond those of conventional training. This difference can reflect the biomechanical characteristics of basketball-specific movements. Actions, such as jumping, sprinting, and sudden stopping, primarily rely on the rapid activation of the plantar flexor muscles (e.g., gastrocnemius, soleus), and PT effectively enhances their eccentric control and concentric explosive strength, leading to concurrent gains in power and functional performance [22]. Conversely, dorsiflexor muscles contribute mainly to gait stability and shock absorption during landing, where conventional training may sufficiently satisfy adaptive needs, explaining the absence of added benefits from PT [23,24]. These findings suggest that PT-induced strength adaptations are task-specific, with improvement magnitudes largely determined by the functional role and force-generating demands of each muscle group during sport-specific actions [25].

Results from the Y-balance test showed that participants in the soft-surface PT group completed the posteromedial and posterolateral directions significantly faster than those in the hard-surface group, and both PT groups outperformed the conventional training group. This indicates that PT-related dynamic balance improvements likely stem primarily from enhanced neuromuscular responsiveness and coordination efficiency; however, since joint mobility was not explicitly measured, its potential contribution cannot be conclusively excluded [26]. Supporting this, subjects showed reduced mediolateral sway and improved stability control during balance tests in the soft-surface group. Stability in the posteromedial and posterolateral directions relies on precise coordination between the ankle plantar flexor–dorsiflexor and hip adductor–abductor muscles. The instability of the soft surface continuously stimulates proprioceptive receptors (e.g., muscle spindles, Golgi tendon organs), promoting faster central nervous system regulation of motor unit recruitment and contraction patterns [20]. This neural adaptation improves movement execution efficiency, reflected by the reduced task completion time. These results align closely with the functional demands of basketball, which require rapid directional changes and sudden stops. Sensor-assisted analyses further showed that participants trained on soft surfaces exhibited faster postural corrections, suggesting improved inter-limb coordination and proprioceptive feedback integration. Compared with completion time, reach distances exhibit no significant group × time interactions. Reach distance reflects maximal functional stability, while the time needed to complete the reaching task indexes movement efficiency, including initiation speed, trajectory smoothness, and the need for corrective adjustments. Thus, soft-surface PT provides a unique advantage in enhancing multidirectional dynamic balance, likely by improving neuromuscular control efficiency and the speed of postural adjustments [27]. These improvements in dynamic balance have direct implications for injury prevention. Previous research indicates that poor performance on the Y-Balance Test is strongly correlated with an elevated risk of lower limb injuries [28].

The static balance results further demonstrated that under the double-leg eyes-closed condition, both the soft- and hard-surface PT groups exhibited significantly smaller AP and ML displacements compared with the conventional training group. Under the single-leg eyes-closed condition, the soft-surface group demonstrated greater improvements in displacement and sway velocity than the hard-surface group, and both groups outperformed the conventional training group. These findings support the theoretical framework that proprioceptive and vestibular compensations are enhanced under visual deprivation [29]. Repetitive proprioceptive stimulation of muscles and joints through PT can promote the central nervous system’s sensitivity to postural perturbations and improve the speed of postural correction [30]. Moreover, the instability of a soft surface amplifies this sensory input, compelling the body to establish a more efficient sensorimotor integration mechanism, and enabling stable posture maintenance even in the absence of visual feedback [31]. Thus, soft-surface PT strengthens balance control under visually deprived conditions, potentially through the optimization of proprioceptive and vestibular compensatory functions. Such an adaptation exhibits high practical relevance for maintaining stability during basketball play, where visual reliance is frequently limited.

Despite the positive short-term benefits observed, several limitations should be acknowledged. The relatively small sample size and short intervention duration may restrict the strength and generalizability of the findings. Meanwhile, the absence of a long-term follow-up assessment prevents us from determining the retention of the observed neuromuscular adaptations, thus limiting practical recommendations for annual training periodization. Because all participants were adolescent female basketball players from a single school, the results should be interpreted within this narrow demographic context and may not extend to male athletes or other age groups. Baseline differences in athletic ability were not fully controlled. Differences in AP and ML sway may relate to underlying biomechanical factors, which were not examined in detail. All participants consisted exclusively of right-leg dominant athletes, which may limit the direct generalizability of the magnitude of effect to left-leg dominant populations, although the underlying physiological mechanisms are presumed to be symmetrical. Future work should include more diverse samples, longer intervention periods, and multi-sensor approaches integrating force sensors, IMUs, EMG, and advanced motion analysis to better elucidate neuromechanical adaptations across different training surfaces.

## 5. Conclusions

The present study demonstrated that PT performed on different support surfaces exerts beneficial effects on balance, explosive power, and muscle strength among adolescent female basketball players with right-leg dominance. In particular, training on a soft support surface elicited greater improvements in static and dynamic balance and enhanced neuromuscular control efficiency compared with training on a hard surface. These results suggest that the unstable properties of a soft surface may promote proprioceptive stimulation and neural adaptation, potentially optimizing intermuscular coordination and postural control.

## Data Availability

The data presented in this study are available on request from the corresponding author due to ethical reasons.

## Abbreviations

The following abbreviations are used in this manuscript:

MVIC	Maximal Voluntary Isometric Contraction
AP	Anteroposterior
ML	Mediolateral
